# A Machine Learning Approach to Pedestrian Detection for Autonomous Vehicles Using High-Definition 3D Range Data

**DOI:** 10.3390/s17010018

**Published:** 2016-12-23

**Authors:** Pedro J. Navarro, Carlos Fernández, Raúl Borraz, Diego Alonso

**Affiliations:** División de Sistemas en Ingeniería Electrónica (DSIE), Universidad Politécnica de Cartagena, Campus Muralla del Mar, s/n, Cartagena 30202, Spain; carlos.fernandez@upct.es (C.F.); raul.borraz@upct.es (R.B.); diego.alonso@upct.es (D.A.)

**Keywords:** pedestrian detection, 3D LIDAR sensor, machine vision and machine learning

## Abstract

This article describes an automated sensor-based system to detect pedestrians in an autonomous vehicle application. Although the vehicle is equipped with a broad set of sensors, the article focuses on the processing of the information generated by a Velodyne HDL-64E LIDAR sensor. The cloud of points generated by the sensor (more than 1 million points per revolution) is processed to detect pedestrians, by selecting cubic shapes and applying machine vision and machine learning algorithms to the XY, XZ, and YZ projections of the points contained in the cube. The work relates an exhaustive analysis of the performance of three different machine learning algorithms: k-Nearest Neighbours (kNN), Naïve Bayes classifier (NBC), and Support Vector Machine (SVM). These algorithms have been trained with 1931 samples. The final performance of the method, measured a real traffic scenery, which contained 16 pedestrians and 469 samples of non-pedestrians, shows sensitivity (81.2%), accuracy (96.2%) and specificity (96.8%).

## 1. Introduction

Autonomous driving is presented as a highly disruptive feature for road means of transport, capable of influencing aspects as fundamental as road safety and mobility itself. Over the last 10 years there have been technological developments which have facilitated the gradual incorporation of various advanced driver assistance systems (ADAS), as well as the emergence of different prototypes capable of travelling in a reasonably autonomous manner.

An autonomous car is a vehicle that is capable of sensing its environment and navigating without human input [1]. Autonomous vehicles promise to radically change both personal and industrial transport. Traffic safety will be improved, since autonomous systems, in contrast to human drivers, have faster reaction times and are fatigue-proof in their functioning. Thus, the number of traffic collisions, dead and injured passengers, and damage to the environment will be reduced. New business models, such as mobility as a service, which aims to be cheaper than owning a car, would be available. Vehicles could be more efficiently used, reducing labour costs for transporting goods and persons (since no driver would be required) and allowing for more pleasant displacements. Finally, thanks to internet connection and vehicular networks, it would be possible to adopt a cooperative approach between vehicles, whereby vehicles and roadside units would share information in peer-to-peer networks. These networks would reduce traffic congestion in cities and roadways, improve safety, and even decrease the need for vehicle insurance. The work [1] reviews this technology, argues the societal benefits it can bring if deployed to the marketplace, and discusses some of the key remaining technology obstacles. Not only big ICT companies like Google are working on this field, but all the major car manufacturers are also investing huge amounts on R+D in order to sell autonomous cars by 2020.

Although some forecasts [2] state that driverless cars will dominate roadways by 2040, the proliferation of fully autonomous vehicles (AV) still seems a distant reality due to technological factors (like the necessity to improve the performance under certain circumstances in order to maximise the safety of the AV in motion, or the necessity to provide security to the computer systems of the AV themselves against potential cyber-attacks), economic factors (like the necessity to reduce the costs of the new technology, or the necessity to adapt road infrastructures to the new vehicles), legal factors (necessity of a new legislation to cover aspects such as insurances, certifications or privacy), as well as sociological factors (like the necessity to face aspects such as habit change, job loss or loss of autonomy) [3].

One of the technological aspects it is necessary to focus on refers to the detection of pedestrians by AVs. Pedestrians represent one of the most unpredictable actors in a scene with road traffic. Thus, more attention is required from situational awareness systems. Identification, tracking, and if possible, movement prediction is basic for avoiding hazards for humans, as well as for increasing the trust of society in AVs. Pedestrian detection has been identified as one of the most important problems AVs face. According to [4], 26,000 people died and 1.4 million were injured in the more than 1 million accidents studied by the European Commission. The situation is more or less similar in the US [5], where 35,000 people died and 2.44 million were injured in the more than 6.3 million accidents reported to the authorities in 2015.

This article describes the Cloud Incubator Car (CIC), an autonomous vehicle based on the Renault Twizy, developed by the División de Sistemas e Ingeniería Electrónica of the Universidad Politécnica de Cartagena, and the sensor system equipped on it for pedestrian detection. Specifically, this article focuses on the HDL-64E 3D Laser Imaging Detection and Ranging (LIDAR) scanner from Velodyne (Morgan Hill, CA, USA), and the machine vision and machine learning algorithms that process the more than one million points per second it generates in order to accurately detect pedestrians around the vehicle.

### 1.1. Pedestrian Detection

Pedestrian detection has been addressed by equipping diverse sensors system on the vehicles and even fusing the data generated by some of them. Cameras and stereoscopic machine vision, laser sensors, time-of-flight cameras, and 3D laser sensors are some examples of the kind of sensors employed to achieve this objective. Despite all the relevant developments in the detection of pedestrians, this task still presents significant challenges. One of them consists in achieving a reliable performance under highly variable lighting conditions, as occur under actual driving conditions.

Pedestrian detection has been studied in the past years, where visual information has been preferentially used to detect them. Some very exhaustive reviews about the current state of the art can be found in [6,7]. The works listed in these articles are mostly related to computer vision methods for detecting pedestrians in images, and they survey the most well-known algorithms and datasets. Normally, pedestrian detection and counting algorithms follow a direct or an indirect approach. Direct approaches rely on pedestrian segmentation and tracking of each individual in the scene, normally requiring strong computer vision algorithms [8]. The indirect approach is map-based, estimating the number of pedestrians in the scene by area [9].

Regarding direct approaches, different visual features have been applied to detect pedestrians, such as the stereoscopic information [10], the movement (e.g., optical flow) [11] or the appearance (e.g., local features like the Histogram of Oriented Gradients [12]). The use of this kind of histograms represents one of the most significant developments to improve algorithms that detect pedestrians. Other kinds of approaches rely on a hierarchical detection structure that combines different types of sensors to improve detection speed. “Fast” sensors are used to detect regions of interest, and then “slow” sensors look for pedestrians in those regions. The work described in [13] employs a low-cost off-the-shelf LIDAR (36° horizontal field of view with 0.08° resolution, 7.125° vertical field of view with 1.425° resolution) sensor to detect regions of interest, a IK-M44H VGA colour camera (Toshiba, San José, CA, USA) to take pictures of only those regions, and a convolutional neural network classifier, in which feature extraction is integrated as a hidden layer of the classifier itself, to confirm whether there is a pedestrian in the region. Although its use demonstrated satisfactory performance on high-quality image sets, the results in actual driving environments are not so satisfactory [6]. The irruption of LIDAR sensors into the market has allowed their use as object detectors. LIDAR sensors are usually made of a set of laser emitters spinning inside a housing. They return range measurements for different horizontal and vertical angles, but since they are made up of a “discrete” number of lasers, the areas in-between the measured points are not sensed. Hence, it is generally not possible to fully reconstruct the underlying geometry of the sensed object. Besides, the environment is measured at different points in time, which hinders even more the processing of the data. Despite the aforementioned disadvantages, LIDAR sensors are being widely used due to their precision in several types of applications, not only autonomous navigation [14], but also subway tunnel model generation [15], precision agriculture [16], and road marking identification [17], to mention just a few.

### 1.2. Pedestrian Detection Using 3D LIDAR Information

The introduction of high–resolution LIDAR 3D sensors has demonstrated the feasibility of this technology to detect pedestrians under actual driving circumstances. One of the first attempts to detect pedestrians by means of LIDAR 3D is described in Premebida’s work [18] and it was performed by using multilayer LIDAR. The author uses a multiple classification method to detect pedestrians in a range of 30 m. He bases his method on the linearity and circularity of 2D data (range data) for feature extraction; by extending this method to 3D data, the computation time increases substantially. Spinello [19] worked afterwards with the 3D point cloud and divides it into various 2D point clouds. These are obtained by cutting the 3D point cloud at different heights, which helps Spinello to determine whether point associations may or may not be part of a pedestrian. This method shows great sensitivity to the viewing distance and is not recommended for ADAS. Navarro-Serment [20] presents a method which extracts information from a point cloud obtained through a high-resolution LIDAR by dividing the cloud into three parts corresponding to the pedestrian’s torso and legs. This process uses the variances of the 3D points of each part as discriminative feature. An advantage that can be highlighted is the low computational load, but it is inconvenient due to the weak behaviour it has when the viewing distance increases. 

A 3D LIDAR developed by the company DENSO (horizontal field of view 40° with 0.1° resolution, vertical field of view 4° with 1° resolution) is described in [21]. The authors also describe a pedestrian detection application using the laser, based on the time-of-flight, intensity and width of the laser signal, and performing a tracking of the pedestrian candidates using an interacting multiple model filter to compute position, velocity and acceleration of the candidates. The authors focus their work on the detection of pedestrians whose trajectory may collide with the car.

More recently, Kidono [22] proposed a method also based on the analysis of a 3D point cloud obtained by means of a high-definition LIDAR. In contrast to the previous authors, Kidono does not subdivide the cloud, but rather makes clusters of points following criteria of height regarding the ground plane and proximity/sparsity among them, and achieves segmenting the various objects in the scenario. The author then classifies the objects which are pedestrians by extracting different features from each portion of segmented points. This method, according to its authors, offers a good recognition rate of pedestrians within a 50 m range.

## 2. Materials and Methods

This section briefly describes the CIC, and the algorithm developed to process the cloud of points generated by the 3D LIDAR in order to detect pedestrians around the vehicle.

### 2.1. CIC Autonomous Vehicle

**Figure 1:**
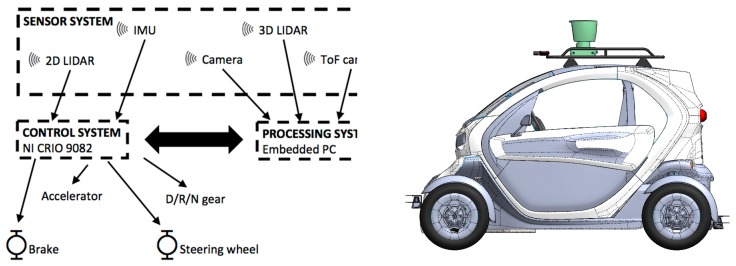
Main components of the CIC architecture.

Figure 1 shows the main components of the autonomous car architecture and their relationship. Each of these parts is described in the following subsections.

#### 2.1.1. Sensor System

The objective of a sensor system is to gather data from the surrounding environment of the AV and feed that data to the control system, where it would be processed and fused to decide what the AV should do next. Assuming that there is no perfect sensor, it is necessary to install multiple sensors in the AV to sense its environment. These sensors measure different physical magnitudes, which are normally selected to overlap with each other, providing the redundant information needed to correctly fuse and correlate the information. This is required to obtain better estimations of the environment, since fusing information gathered from different sensors helps reducing the number of plausible interpretations of the measured data in dynamic and unknown environments. Hence, the selection of sensors types and their placement are highly important.

**Figure 2:**
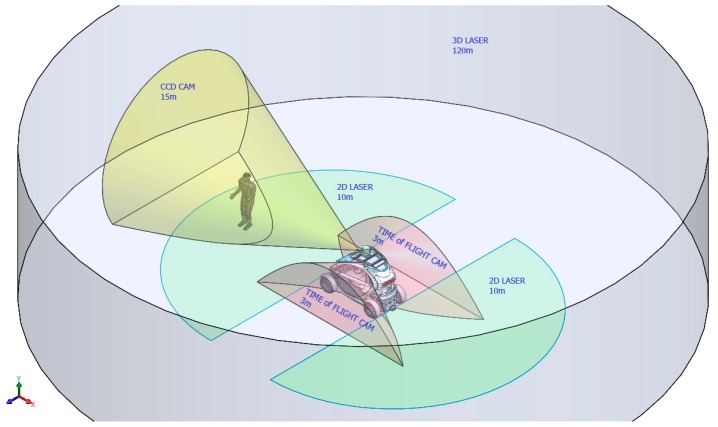
Range diagram of the sensors mounted on the CIC.

Figure 2 shows the areas around the CIC covered by each sensor mounted on it. Two kind of sensors are used for measuring the environment: short range sensors (up to 10 m) and long range sensors. The installed short range sensors include a 2D Sick laser range scanner and a time-of-flight camera. The long range sensors are a 3D LIDAR scanner and a camera in the visible spectrum.

**Figure 3:**
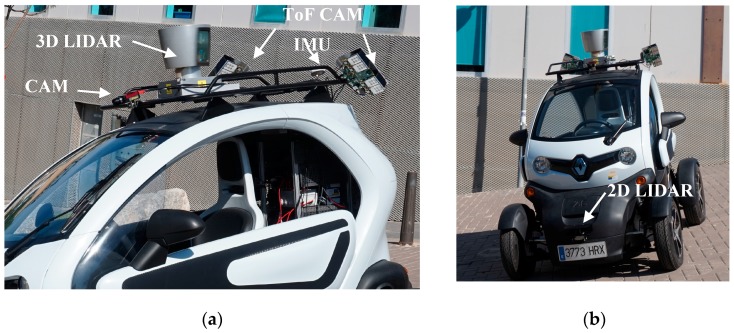
Sensor system on-board the car roof (**a**), and front (**b**) of the CIC.

**Short-range sensors.** The car carries two TIM55x Laser scanners (Sick, Waldkirch, Germany, see Figure 3b), one on the front of the vehicle and one on the back, parallel to the ground and at a height of 30 cm. Each laser covers 180° with an angular resolution of 1°, has a scanning range of 0.05 to 25 m with a systematic error of ±60 mm, and spins at 15 Hz. The lasers are connected to the central control system through an Ethernet connection. The vehicle also carries two SENTIS3D-M420 time-of-flight cameras (BlueTechnix, Wien, Austria) one on each side of the vehicle (see Figure 3a). Their purpose is to gather data from the closest surroundings, which are too close for the 3D LIDAR. The effective operating distance is limited to approximately 4 m. Each camera captures up to 160 images per second, with 160 × 120 pixels resolution, independently of the outside lighting conditions.

**Long-range sensors.** The vehicle is equipped with a camera with a 1.2 Mpixels Charge-Coupled Device (CCD) in the visible spectrum, a Prosilica GT1290 from Allied Vision (Stadtroda, Germany), with a Gigabit Ethernet connection. It can capture up to 33 images per second, in grayscale or colour (see Figure 3a). CIC also has a Velodyne HDL-64E, a high-definition 3D LIDAR scanner (see Figure 3a) with 64 laser sensors. The LIDAR spins from 5 to 20 Hz, covering the complete 360° horizontally (from 0.086° to 0.345° angular resolution) and 26.8° vertically (separation between laser sensors 0.4°). The accuracy of the distance measurement is less than 2 cm, and maximum range is between 50 m (low reflective surfaces, like pavement) and 120 m (high reflective surfaces). Minimum distance is 0.9 m. The LIDAR produces more than 1 million points per second, reporting for each point its distance and the measured reflectivity. Depending on the revolution speed, it can capture from 66,000 to 266,000 points per revolution (Figure 3a).

#### 2.1.2. Control System

**Figure 4:**
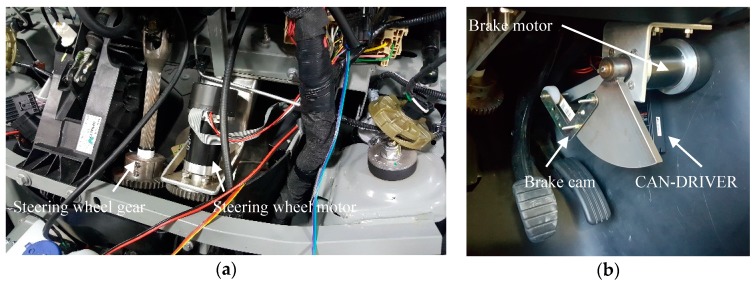
Actuation system: (**a**) steering wheel and (**b**) breaking pedal.

The main control systems of the Renault Twizy have been automated in order to allow the vehicle to be autonomously controlled. The modified systems are the steering wheel (see Figure 4a), the brake pedal (see Figure 4b), and the accelerator pedal. For the steering wheel, we have installed a gear and a clutch in the steering axis, so that we can rotate it by using an electrical motor. The brake pedal is actioned mechanically by a cam controlled by an electric motor, with a maximum rotation angle of 15°. The accelerator pedal is electronically simulated by the control system, which emulates the electronic analog signals generated by the real pedal. Both motors (steering wheel and brake pedal) are controlled by two controller drives through a CAN bus.

**Figure 5:**
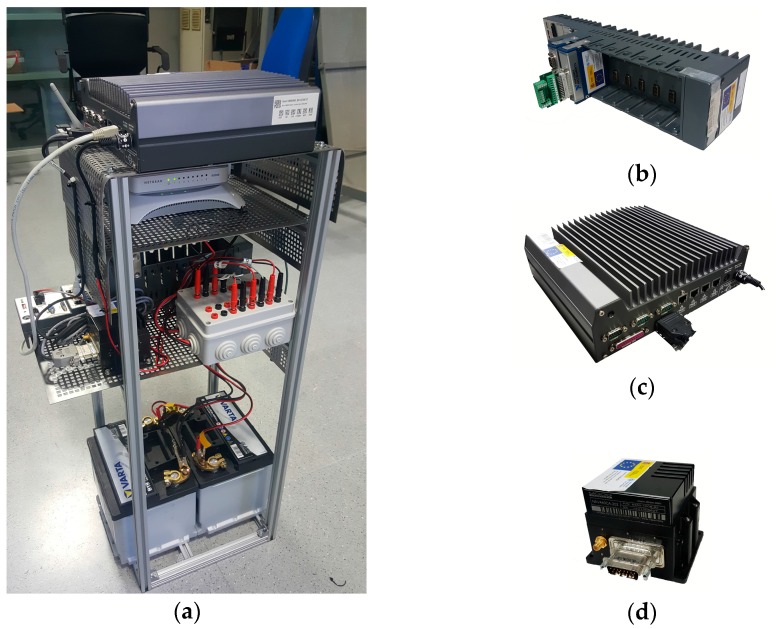
Control system: (**a**) aluminium structure; (**b**) cRIO 9082; (**c**) Emmbebed PC; (**d**) IMU.

The hardware of the control system, installed inside the back of the car in an aluminium structure (see Figure 5a), comprises two 12 V DC batteries to isolate our systems from the car power system, a Compact-Rio 9082 Controller (National Instruments, Austin, TX, USA, NI cRIO 9082, see Figure 5b), a NAV440CA-202 Inertial Measurement Unit (IMU, Moog Crossbow, Milpitas, CA, USA, see Figure 5d), and lastly, a 3G WIFI router modem and Gigabit Ethernet switch. The cRIO 9082 controls the accelerator, brake and steering wheel movements with the CAN-Open communication protocol, as well as I/O signals.

#### 2.1.3. Processing System

The main element of the processing system is a Nuvo-1300S/DIO computer (Neousys, New Taipei City, Taiwan, see Figure 5c), a fan-less embedded controller featuring an Intel Core i7 processor and 4 GB of RAM. The processing system carries out the high performance tasks associated with CIC navigation, such as: (1) execution of computer vision algorithms; (2) processing the cloud of points generated by the 3D LIDAR, to detect pedestrians, other vehicles, curbstones and so on; (3) generation of new trajectories and execution of obstacle avoidance manoeuvres; (4) test the correct operation of the rest of the systems; and (5) storing the data of the sensors on disk for later off-line analysis.

### 2.2. 3D LIDAR Cloud of Points

The Velodyne HDL-64E sends the information (both distance to the point and reflectivity value) through UDP packets, configured to broadcast on port 2368. Each packet contains a data payload of 1206 bytes, comprising 12 blocks of 100 bytes of laser data, plus 6 bytes of calibration and other information pertaining to the sensor. Each block contains data from 32 lasers (three bytes per laser), plus information regarding the laser block number (two bytes) and the azimuthal angle (two bytes). Azimuthal angle is reported from 0 to 35,999 (tenths of a degree). Regarding the measurements reported by each laser sensor, two bytes encode distance to the nearest 0.2 cm, and the remaining byte reports intensity on a scale of 0–255. Six firings of each of the laser blocks takes 139 µs, plus around 100 µs to transmit the entire 1248 byte UDP packet, which equals 12.48 bytes/µs. Data points can be transformed to a Cartesian coordinates system by applying the equations listed in (1), where α is the azimuthal angle, and ω is the vertical angle:

(1) $$\begin{array}{l} x=distance\cdot \text{cos}\left(\omega \right)\cdot \text{sin}\left(\alpha \right)\\ y=distance\cdot \text{cos}\left(\omega \right)\cdot \text{cos}\left(\alpha \right)\\ z=distance\cdot \text{sin}\left(\omega \right) \end{array}$$

Distance measures are collected by the LIDAR, and organized in a cloud of points covering the complete 360° around the vehicle. At its current rotation speed (10 Hz), the LIDAR produces more than 1 million points per revolution, with an angular resolution of 0.1728°. This laser data is barely pre-processed: only values below the minimum (lower than 500) are deleted from the cloud of points before it is passed to the pedestrian detection algorithm, described in the following section.

### 2.3. Pedestrian Detection Algorithm

The pedestrian detection algorithm described below revolves around the processing of clusters of points contained inside a cube of dimensions 100 × 100 × 200 cm (the size of a human being). The algorithm comprises the following five steps:

1. Select those cubes that contain a certain number of points, inside a threshold. This allows to eliminate objects with a reduced number of points which could produce false positives. Also, it reduces the computing time.
2. Generate XY, YZ and XZ axonometric projections of the points contained inside the cube.
3. Generate binary images of each projection, and then pre-process them.
4. Extract features from each axonometric projection and 3D LIDAR raw data.
5. Send the feature vector to a machine learning algorithm to decide whether it is a pedestrian or not.

**Figure 6:**
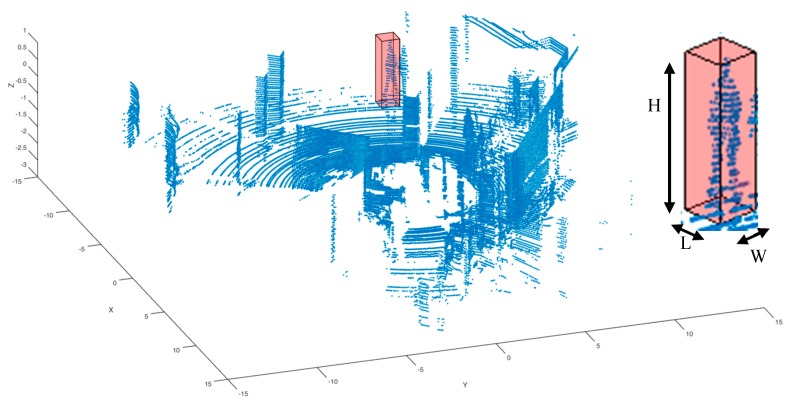
Pedestrian sample captured with software tools over a frame from the 3D LIDAR.

Figure 6 shows a screen-shot of all the data gathered by the Velodyne HDL-64 LIDAR in one revolution. We have highlighted inside a red cube one person, so that the reader can better understand the following steps. Cube selection is the first step of the algorithm, and it’s very simple. The threshold for applying the algorithm described below to the cube is between 150 and 4000 points, and these numbers have been empirically determined by analysing three thousand frames captured by the LIDAR.

#### 2.3.1. Step 2: Generation of XY, YZ and XZ Axonometric Projections 

XY, XZ and YZ axonometric projections are calculated from a sample that has enough points. The sample is normalized in function of its maximum and minimum local values of each coordinate X, Y, Z according to the equations listed in (2):

(2) $$\begin{array}{l} X_{\text{norm}}^{\left(n\right)}=\frac{X^{\left(n\right)}-\text{min}\left(X^{\left(n\right)}\right)}{\text{max}\left(X^{\left(n\right)}\right)-\text{min}\left(X^{\left(n\right)}\right)}\\ Y_{\text{norm}}^{\left(n\right)}=\frac{Y^{\left(n\right)}-\text{min}\left(Y^{\left(n\right)}\right)}{\text{max}\left(Y^{\left(n\right)}\right)-\text{min}\left(Y^{\left(n\right)}\right)}\\ Z_{\text{norm}}^{\left(n\right)}=\frac{Z^{\left(n\right)}-\text{min}\left(Z^{\left(n\right)}\right)}{\text{max}\left(Z^{\left(n\right)}\right)-\text{min}\left(Z^{\left(n\right)}\right)} \end{array}$$

Being ‘*n*’ the number of the sample, and ‘*ns*’ the number of points in the sample.

**Figure 7:**
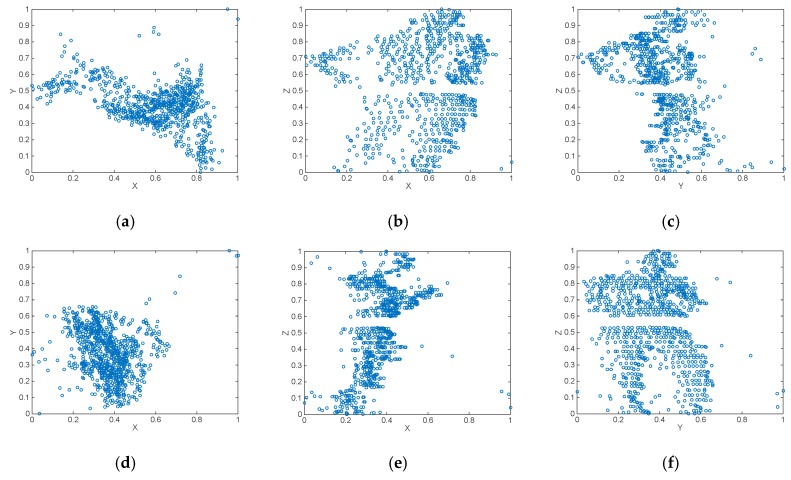
Normalized XY, XZ and YZ axonometric projections of two pedestrian samples: (**a**–**c**) pedestrian one; (**d**–**f**) pedestrian two. By applying different coefficients to the normalized data, while keeping the aforementioned proportions, we can generate several binary images. But these images are only composed of dots (the points where a laser beam hit an object), see Figure 8a–f.

$X^{\left(n\right)}=\left\{x_{1},\ldots ,x_{ns}\right\},\;Y^{\left(n\right)}=\left\{y_{1},\ldots ,y_{ns}\right\},\;Z^{\left(n\right)}=\left\{z_{1},\ldots ,z_{ns}\right\}$ are the sets of Cartesian coordinates of all points contained inside the sample. After normalization, the new sets $\left\{X_{\text{norm}}^{\left(n\right)},\;Y_{\text{norm}}^{\left(n\right)},\;Z_{\text{norm}}^{\left(n\right)}\right\}$ values are placed in the interval (0,1). Figure 7 shows the normalized axonometric projections of two pedestrian samples.

#### 2.3.2. Step 3: Generation and Pre-processing of Binary Images of Axonometric Projections

We now need to generate an image-like artefact from the normalized values obtained in step 2, so that we can afterwards apply computer vision algorithms to each of the images generated from the axonometric projections. We tackle two related problems: how to generate an image from the normalized values in a way that keeps the original proportions to avoid deformations, and in a way that eases further processing. We aim to generate binary images, where the background is set to black (value ‘0’), and the pixels that correspond to points of the cloud are set to white (value ‘1’). Since the cloud of points being processed is obtained from a cubic region of size 100 × 100 × 200 cm, the proportions of the scaling coefficients that we have applied to the normalized values obtained in step 2 are 1-1-2.

**Figure 8:**
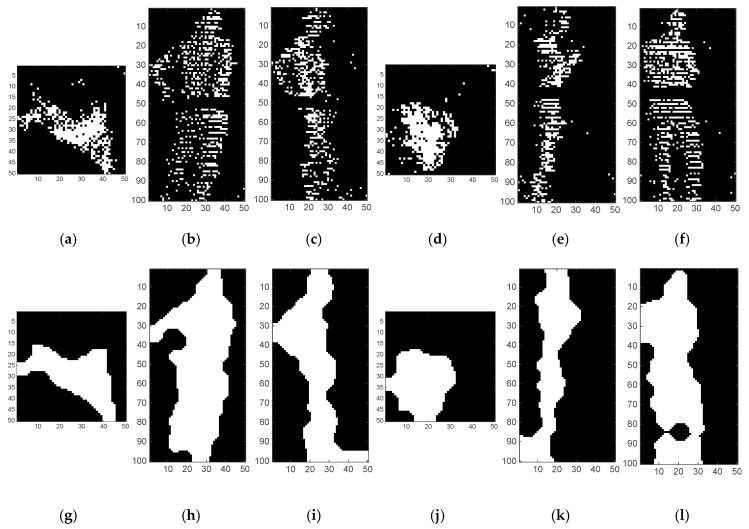
(**a**–**f**) binary images generated from the XY, XZ and YZ projections of two pedestrian samples; (**g**–**l**) results of the pre-processing of the binary images.

We need to further process the images in order to obtain the “real” object was that generated by the cloud of points being analysed. For this, we apply several algorithms to remove small particles, reduce noise, smooth objects, and obtain a compact figure of the object to be recognized afterwards by the machine learning algorithm, as shown in Figure 8g–l. For this purpose, we apply two morphological operations and one filter operation in the following order: (1) an opening operation to achieve a compact set of pixels; (2) a filter to erase small particles; and (3) a closing operation to smooth edges.

The values we select for all operations are strongly related: if the images we generate are big, the processing algorithms have to be very aggressive to generate compact images. But if the images are small, we can lose information in the generation of the binary images. We empirically obtained the following values. *X* and *Y* axes have been mapped to 50 pixels, while the *Z* axis has been mapped to 100 pixels. These rates avoid the apparition of holes in the images and reduces the pre-processing. We used the ‘ceil’ operation to round values, since the image’s pixels start at value ‘1’. The opening operation was configured to use a radius of six pixels, the filter erases particles with areas of less than 200 pixels, and the closing operation was configured to use a ratio of three pixels. Figure 8a–f shows the binary images obtained from the samples are shown in Figure 7, while Figure 8g–l shows the results of the pre-processing stage with these values.

#### 2.3.3. Step 4: Compute Features Vector

In machine learning or computer vision, a feature vector is a n-dimensional vector of numerical features that represent an object [23]. The size of the vector and the features it stores depends on the objects you want to search for, and on the conditions the data was gathered. In order to increase or highlight the raw data, transformations to spaces such as Fourier [24], Gabor [25] or Wavelet [26,27] are commonly used. In other cases, raw data is reduced to binary format (e.g., grayscale images to binary images) in order to find geometric features of object [28], such as area, perimeter, compacity, excentricity, etc., which will allow the shape of the object to be characterized. The statistical features of first and second order [29,30] have also been often used as features in classifiers and machine learning systems [31].

In order to separate pedestrians from the rest of the information contained in the cloud of points, we compute a feature vector composed of fifty features (f_1_, …, f_50_) per projection. The features can be divided into three groups. The first group measures shape characteristics. The second group measures the seven Hu invariant moments [32,33]. The third group computes statistical information, specifically, the normalized distance and the reflexivity reported from the 3D LIDAR raw data. Table 1 shows a summary of the fifty features contained in the vector, according to the group they belong to. The shape elements of the feature vector are:

1. Area: number of pixels of the object.
2. Perimeter: specifies the distance around the boundary of the region.
3. Solidity: proportion of the pixels in the convex hull that are also in the region.
4. Equivalent diameter: Specifies the diameter of a circle with the same area as the object (see Equation (3)):
  (3) $$ED=\sqrt{\frac{4*\text{Area}}{\pi }}$$
5. Eccentricity: Ratio of the distance between the foci of the ellipse and its major axis length. The value is between 0 and 1.
6. Length of major/minor axis. A scalar that specifies the length (in pixels) of the major/minor axis of the ellipse that has the same normalized second central moments as the region under consideration.

The Hu invariant moments with respect to translation, scale and rotation are computed as shown in Equations (4)–(10):

(4) $$M_{1}=\left(\eta_{20}+\eta_{02}\right)$$

(5) $$M_{2}={\left(\eta_{20}-\eta_{02}\right)}^{2}+4\eta_{11}^{2}$$

(6) $$M_{3}={\left(\eta_{30}-3\eta_{12}\right)}^{2}+{\left(3\eta_{21}-\eta_{03}\right)}^{2}$$

(7) $$M_{4}={\left(\eta_{30}+\eta_{12}\right)}^{2}+{\left(\eta_{21}+\eta_{03}\right)}^{2}$$

(8) $$\begin{array}{c} M_{5}=\left(\eta_{30}-3\eta_{12}\right)\left(\eta_{30}+\eta_{12}\right)[{\left(\eta_{30}+\eta_{12}\right)}^{2}-3{\left(\eta_{21}+\eta_{03}\right)}^{2}]\\ +\left(3\eta_{21}-\eta_{03}\right)\left(\eta_{21}+\eta_{03}\right)[3{\left(\eta_{30}+\eta_{12}\right)}^{2}-{\left(\eta_{21}+\eta_{03}\right)}^{2}] \end{array}$$

(9) $$\begin{array}{l} M_{6}=\left(\eta_{20}-\eta_{02}\right)[{\left(\eta_{30}+\eta_{12}\right)}^{2}-{\left(\eta_{21}+\eta_{03}\right)}^{2}]\\ +4\eta_{11}\left(\eta_{30}+\eta_{12}\right)(\eta_{21}+\eta_{03}) \end{array}$$

(10) $$\begin{array}{l} M_{7}=\left(3\eta_{21}-\eta_{03}\right)\left(\eta_{21}+\eta_{03}\right)[3{\left(\eta_{30}+\eta_{12}\right)}^{2}-{\left(\eta_{21}+\eta_{03}\right)}^{2}]\\ -\left(\eta_{30}-3\eta_{12}\right)\left(\eta_{21}+\eta_{03}\right)[3{\left(\eta_{30}+\eta_{12}\right)}^{2}-{\left(\eta_{21}+\eta_{03}\right)}^{2}] \end{array}$$

where ‘$\eta_{pq}$’ is the normalized central moment of order (p + q), computed as shown in Equation (11):

(11) $$\eta_{pq}=\frac{\mu_{pq}}{\mu_{00}^{w}},\;w=\frac{p+q}{2}+1,\forall \;p+q\geq 2$$

The statistical elements of the feature vector are: mean, standard deviation, kurtosis, and skewness (see Equations (12)–(15)). These values are computed over normalized distance and reflexivity from 3D LIDAR raw data, generating eight features. Normalised distance is computed as shown in Equation (2):

(12) $$\begin{array}{ccc} \mu_{ND}^{\left(n\right)}=\frac{1}{ns}\overset{ns}{\underset{i=1}{\sum }}ND_{i}, & & \mu_{R}^{\left(n\right)}=\frac{1}{ns}\overset{ns}{\underset{i=1}{\sum }}R_{i} \end{array}$$

(13) $$\begin{array}{ccc} s_{ND}^{\left(n\right)}=\sqrt{\frac{1}{ns}\overset{ns}{\underset{i=1}{\sum }}{\left(ND_{i}-\mu_{ND}^{\left(n\right)}\right)}^{2}}, & & s_{R}^{\left(n\right)}=\sqrt{\frac{1}{ns}\overset{ns}{\underset{i=1}{\sum }}{\left(R_{i}-\mu_{R}^{\left(n\right)}\right)}^{2}} \end{array}$$

(14) $$\begin{array}{ccc} k_{ND}^{\left(n\right)}=\frac{\frac{1}{ns}\sum_{i=1}^{ns}{\left(ND_{i}-\mu_{ND}^{\left(n\right)}\right)}^{4}}{{\left(s_{ND}^{\left(n\right)}\right)}^{4}}, & & k_{R}^{\left(n\right)}=\frac{\frac{1}{ns}\sum_{i=1}^{ns}{\left(R_{i}-\mu_{R}^{\left(n\right)}\right)}^{4}}{{\left(s_{R}^{\left(n\right)}\right)}^{4}} \end{array}$$

(15) $$\begin{array}{ccc} sk_{ND}^{\left(n\right)}=\frac{\frac{1}{ns}\sum_{i=1}^{ns}{\left(ND_{i}-\mu_{ND}^{\left(n\right)}\right)}^{3}}{{\left(s_{ND}^{\left(n\right)}\right)}^{3}}, & & sk_{R}^{\left(n\right)}=\frac{\frac{1}{ns}\sum_{i=1}^{ns}{\left(R_{i}-\mu_{R}^{\left(n\right)}\right)}^{3}}{{\left(s_{R}^{\left(n\right)}\right)}^{3}} \end{array}$$

Being $\mu_{ND}^{\left(n\right)}$, $\mu_{R}^{\left(n\right)}$, $s_{ND}^{\left(n\right)}$, $s_{R}^{\left(n\right)}$, $k_{ND}^{\left(n\right)}$, $k_{R}^{\left(n\right)}$, $sk_{ND}^{\left(n\right)}$ and $sk_{R}^{\left(n\right)}$ the means, standard deviations, kurtosis, and skewness of the set of ‘*ns*’ laser measurements of normalized distance ($ND_{i}$) and reflexivity ($R_{i}$) of a sample ‘*n*’.

#### 2.3.4. Step 5: Machine Learning Algorithm

We are in the Big Data era. Sixty trillion web pages are added every day [34]; one hour of video is uploaded to YouTube every second; new 3D LIDAR sensors supply four billion points per hour. Machine learning (ML) technics offer solutions to automate big data analysis. A short definition of ML is that it is a set of methods that can automatically detect patterns in data. Then, we can make use of this uncovered patterns to make predictions, or to perform other kinds of decision making under uncertainty [35]. ML methods are usually classified into two types: supervised and unsupervised learning.

Supervised learning is based on a labelled training data set, which is used to extract a mathematical model of the data. Supervised methods use Euclidean distance, Bayes theorem, linear regression, Gaussian distributions and so on to create the models. Among the most well-known supervised methods are k-Nearest Neighbours, Naïve Bayes classifiers, Artificial Neural Networks, or Support Vector Machines [27].

Unsupervised learning doesn’t use a labelled training dataset because it doesn’t try to learn anything in particular. Unsupervised learning divides the dataset into homogeneous groups, which is called clustering. It is used quite regularly for data mining. For instance, it can be used to detect patterns of fraudulent behaviour or in market stock analysis. K-means, mixture models or hierarchical clustering are approaches to unsupervised learning [36].

In this work, we tested the performance of three ML Algorithms (MLA) to detect pedestrians: k-Nearest Neighbours (kNN), Naïve Bayes Classifier (NBC), and Support Vector Machine (SVM). These algorithms were used to implement binary classifiers to detect two kinds of objects, pedestrian and no pedestrian, and they were tested with different configuration parameters and kernel functions (see Table 2). kNN was tested with Euclidean and Mahalanobis distance with data normalization; NBC was tested with Gauss and Kernel Smoothing Functions (KSF) without data normalization; and SVM was tested with linear and quadratic functions with data normalisation. 

The reasons to select these MLA are: (1) they are easy to implement and reproduce the results, since they can be found in most MLAs libraries and programming languages such as: MATLAB, R, Python, C++, etc.; (2) they are very popular, we can find lots of works that use them, and we can compare them; (3) they are easy to configure and test and (4) in our experience [27,37], they produce good results.

## 3. Results and Discussion

**Figure 9:**
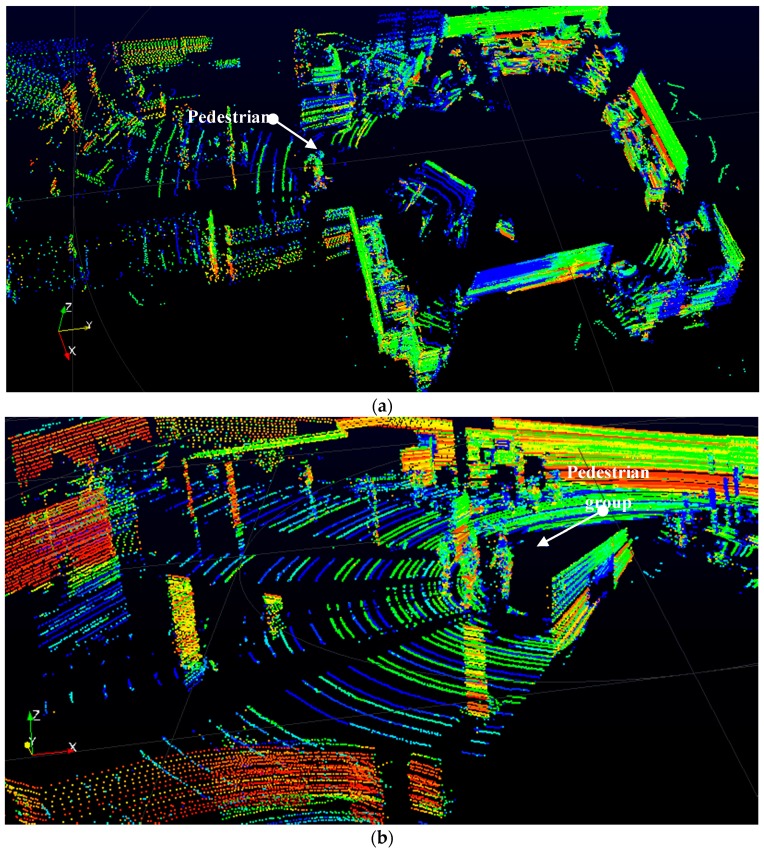

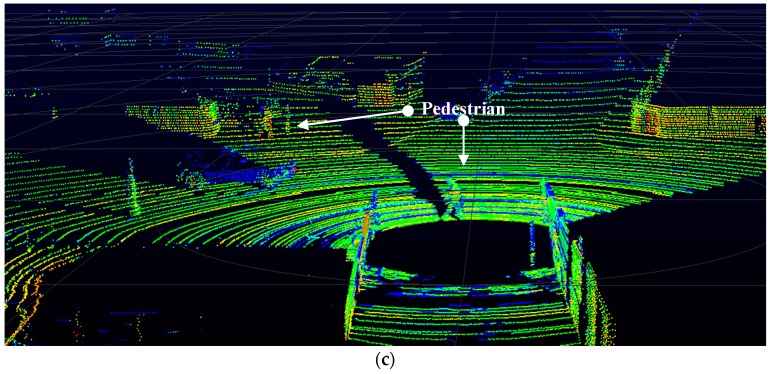
Three scene frames: (**a**) Intelligent Vehicles and Computer Vision Lab; (**b**) underground parking; (**c**) Real traffic scene. The colour of the points in the images represents the reflexivity value reported by the 3D LIDAR.

Since the proposed algorithm for pedestrian detection comprises a MLA, and we have tested the performance of kNN, NBC, and SVM algorithms, we divide this section into two parts: performance and selection of the MLA that will detect pedestrians in the cloud of points generated by the 3D LIDAR, and performance of the overall algorithm in a real scenario. For this purpose, we have defined three scenarios. The first and second scenarios have been used to extract a set of samples of pedestrian and no-pedestrian objects from the data generated by the 3D LIDAR. The first scenario corresponds to the Intelligent Vehicle and Computer Vision Laboratory of our research group (DSIE). In this scenario, samples have been taken of a pedestrian in different angles and distances, as well as other elements such as walls, obstacles, vehicles, etc. (see Figure 9a). The second scenario is an underground parking, and it was used to capture pedestrians and multiple samples of cars, motorbikes, columns, and so on (see Figure 9b). The third scenario corresponds to a real traffic area around the Polytechnic University of Cartagena (see Figure 9c). This last scenario was used to test the system in a real traffic area.

### 3.1. Performance of Machine Learning Algorithms

Before selecting the MLA for pedestrian detection, we first need a training set. To capture a sample of points representative of a pedestrian, among the more than 1 million points generated by the 3D LIDAR in every revolution (or frame), we developed a software tool in MATLAB. This tool, shown in Figure 6, loads previously stored laser data and allows users to select sample s of points of various sizes. Default values of the sample are set to 100 × 100 × 200 centimetres. Users can also rotate the frame, filter points according to their distance to the origin of coordinates, as well as modify the size of the selection sample. Once a sample is selected, the software automatically stores the raw data from the sample in a raw feature vector, which includes X, Y, Z coordinates, distance and reflexivity, and allows the user to state whether the sample contains a pedestrian or not (i.e., its label). From the first and second scenarios, we have extracted with the MATLAB tool 277 pedestrians and 1654 no-pedestrian samples. There are several evaluation metrics that can be used to evaluate the performance of MLA: calculate values based on the confusion matrix, generate the Receiver Operating Characteristic (ROC) curve, or perform the leave-one-out cross-validation method (LOOCV). We present the results we have obtained for all considered MLA in this section. LOOCV and ROC curves have been computed under different configurations of the MLAs, as shown in Table 3. The table also shows the errors obtained after applying LOOCV and ROC curves analysis to the training set. These metrics allowed us to select the optimal MLA for the last step of the pedestrian detection algorithm described in this article.

The confusion matrix presents the number of samples that were correctly classified by a MLA (true positives (TP) and true negatives (TN)) against those which weren’t (false positives (FP) and false negatives (FN)). In the case of binary classifiers, such as the one we propose in this article to detect pedestrians, we can compute several values from the confusion matrix, such as accuracy (Equation (19), the proportion of correctly classified samples); precision (Equation (18), assesses the predictive power of the algorithm); sensitivity/specificity (Equations (16) and (17), assesses the effectiveness of the algorithm on detecting a single class); and F-score (Equation (20), the harmonic mean of precision and sensitivity). These are normally the first metrics considered when evaluating a MLA, specially accuracy. However, these numbers alone cannot fully capture the performance of an MLA, in the same way as the mean does not capture all the characteristics of a data set. 

**Figure 10:**
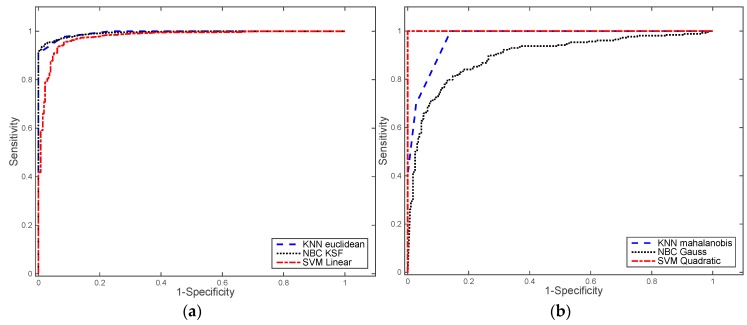
ROC results: (**a**) kNN classifier with Euclidean distance, NBC with KSF, and SVM classifier with linear functions; (**b**) kNN classifier with Mahalanobis distance, NBC with Gauss kernel, and SVM classifier with quadratic polynomial function.

The ROC curve better characterises the performance of MLAs. It is calculated by comparing the rate of true positives (sensitivity, see Equation (16)) against the rate of false positives (1-specificity, see Equation (17)) at various threshold levels. The Area Under the Curve (AUC) is also an important value, since it is a measure of the discrimination power of the classifier, that is, the ability of the classifier to correctly classify the patterns submitted (pedestrian and non-pedestrian in our case) [38,39]. AUC can also be interpreted as the probability that the classifier will assign a higher score to a randomly chosen positive example than to a randomly chosen negative example. Figure 10 shows ROC curves computed for each MLA with different kernel functions:

(16) $$Sensitivity\;or\;Recall=\frac{TP}{TP+FN}$$

(17) $$Specificity=\frac{TN}{TN+FP}$$

(18) $$Precision=\frac{TP}{TP+FP}$$

(19) $$Accuracy=\frac{\left(TP+TN\right)}{\left(TP+FP+FN+TN\right)}$$

(20) $$Fscore=\frac{2*TP}{2*TP+FP+FN}$$

LOOCV is a simple cross-validation method, which extracts one sample of the training set and uses the remaining samples for training the MLA. After that, it classifies the sample it took apart and computes the error of the classification. This process is repeated for all samples. The LOOCV error is afterwards computed as the mean of the errors of the classification of each sample on its own, representing a measure of how well the model adjusts itself to the supplied data. LOOCV is a method that is computationally expensive. Both LOOCV and AUC analysis shows that the best fitting of data model is produced by SVM with quadratic kernel, and the worst results were obtained with the NBC with Gaussian kernel. 

### 3.2. Performance of the Complete Pedestrian Detection Algorithm

To determine the best MLA to be used in the pedestrian detection algorithm, we selected and tested in scenario 3 (see Figure 9c) the algorithms that had the lower LOOCV error, the higher AUC, and the higher Fscore in Table 3. Thus, we selected kNN with Euclidean distance and SVM with linear and quadratic functions. From scenario 3, we have been selected seven frames of thirty square meters, and all of them have been partitioned in samples of 100 × 100 × 200 cm. Table 4 shows the results of the performance of the selected MLAs, obtained after a manual computation of the TP, FP, TN and FN. The number of pedestrian in each frame has been determined manually (see Table 4 third column).

Table 5 shows the final metric results of the tested MLAs. As shown in the table, the best compromise between the metrics computed corresponds with SVM with linear function, which is the MLA we finally selected.

The differences we find between the theoretical results of Table 3 (where SVM with a quadratic kernel shows the best results) with respect to the results of scenario 3 (shown in Table 5), are due to the “overfitting problem”. A MLA presents overfitting when it is capable of learning all of the samples provided in the training set by itself, but it is not able to generalize to new data. Given the results shown in Table 3, we expected SVM with quadratic kernel to achieve a 100% success when classifying new, unknown samples, but its performance decreases in all metrics, as shown in the last column of Table 5, being even lower than other MLAs.

The overfitting problem can be caused by many factors, one of which is a low number of training samples. In our case, we tried a different number of training samples (from 200 to 2000 with step of 200), but the problem persisted. For this reason, we finally decided to use the linear SVM as MLA for pedestrian detection.

**Figure 11:**
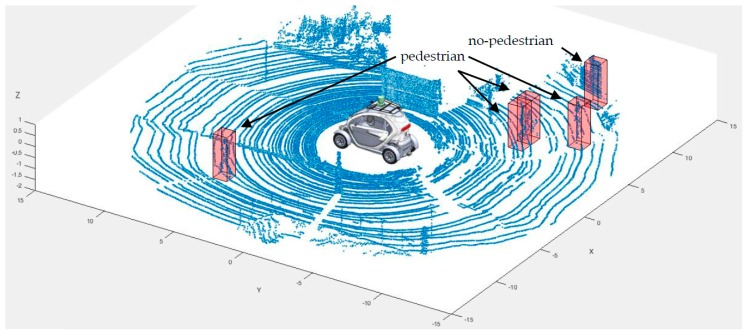
Pedestrian detection algorithm on real traffic area.

Figure 11 shows the result obtained to execute the pedestrian detection algorithm over frame six, where there are three pedestrians. In this frame, the algorithm evaluated forty-five samples. As shown in the picture, the algorithm detected four pedestrians (one false positive).

Table 6 shows a comparison of the proposed method with the five methods we reviewed in Section 1.2, which use High Definition 3D LIDAR to carry-out pedestrian detection.

The comparison is made in terms of MLA implementation, metric used (graphical and numeric values) and performance. We want to highlight that there is a lack of homogeneity in the reporting of performance statistics in the works reviewed, and an absence of numerical results in many of them. This fact makes performing a thorough comparison of the results almost impossible. Nevertheless, we have estimated the AUC of the works [20,22], and use the Fscore metric (see Equation (20)) to compare with the works where the authors report ad-hoc mean values, since Fscore is defined as the harmonic mean of precision and sensitivity (or recall) and it establishes the relationship between them. As shown in Table 6, the AUC of authors [20,22] is lower than the proposed method and the performance mean reported by [18,19,21] is less than the Fscore obtained by the proposed method.

## 4. Conclusions and Future Work

This article presents a machine learning approach to pedestrian detection for autonomous vehicles using high-definition 3D range data, since pedestrian detection has been identified as one of the most critical tasks for this kind of vehicles. The work is included in the development of an autonomous vehicle based on a Renault Twizy platform by the DSIE Research Group at UPCT. The major remarkable features of the proposed approach are:

- Unlike other works [18,20,22], a novel set of elements is used to construct the feature vector the machine learning algorithm will use to discriminate pedestrians from other objects in its surroundings. Axonometric projections of cloud points samples are used to create binary images, which has allowed us to employ computer vision algorithms to compute part of the feature vector. The feature vector is composed of three kind of features: shape features, invariant moments and statistical features of distances and reflexivity from the 3D LIDAR.
- An exhaustive analysis of the performance of three different machine learning algorithms have been carried out: k-Nearest Neighbours (kNN), Naïve Bayes classifier (NBC), and Support Vector Machine (SVM). Each algorithm was trained with a training set comprising tool 277 pedestrians and 1654 no pedestrian samples and different kernel functions: kNN with Euclidean and Mahalanobis distances, NBC with Gauss and KSF functions and SVM with linear and quadratic functions. LOOCV and ROC analysis were used to detect the best algorithm to be used for pedestrian detection. The proposed algorithm has been tested in real traffic scenarios with 16 samples of pedestrians and 469 samples of non-pedestrians. The results obtained were used to validate theoretical results obtained in the Table 3. An overfitting problem in the SVM with quadratic kernel was found. Finally, SVM with linear function was selected since it offers the best results.
- A comparison of the proposed method with five other works that also use High Definition 3D LIDAR to carry-out the pedestrian detection, comparing the AUC and Fscore metrics. We can conclude that the proposed method obtains better performance results in every case.

Pedestrian detection has traditionally been performed using machine vision and cameras, but these techniques are affected by changing lighting conditions. 3D LIDAR technology provides more accurate data (more than 1 million points per revolution), which can be successfully used to detect pedestrians in any kind of lighting conditions.

The high success rate and scalability of machine learning algorithms will enable the detection of different objects during vehicle navigation. The authors are working on increasing the data base of point cloud samples, as well as on creating learning machines capable of detecting other important objects in the scene, such as bikes, cars, traffic signs and lights, etc. Sensor fusion is also very important, and therefore we are very interested in using the information from the cameras mounted on our car. Also, given that the algorithms are computationally expensive, the authors are developing parallelizable code to increase the speed of the algorithm. 

## Figures and Tables

**Table 1:** Feature vector composition.

Shape Features	Invariant Moments	Statistical Features
f_1_, f_2_, f_3_: Areas of XY, XZ, YZ projections	f_22_, f_23_, f_24_: Hu moment 1 over XY, XZ, YZ projections	f_43_, f_44_: Means of distances and reflexivity
f_4_, f_5_, f_6_: Perimeters of XY, XZ, YZ projections	f_25_, f_26_, f_27_: Hu moment 2 over XY, XZ, YZ projections	f_45_, f_46_: Standard deviations of distances and reflexivity
f_7_, f_8_, f_9_: Solidity of XY, XZ, YZ projections	f_28_, f_29_, f_30_: Hu moment 3 over XY, XZ, YZ projections	f_47_, f_48_: Kurtosis of distances and reflexivity
f_10_, f_11_, f_12_: Equivalent diameters of XY, XZ, YZ projections	f_31_, f_32_, f_33_: Hu moment 4 over XY, XZ, YZ projections	f_49_, f_50_: Skewness of distances and reflexivity
f_13_, f_14_, f_15_: Eccentricity of XY, XZ, YZ projections	f_34_, f_35_, f_36_: Hu moment 5 over XY, XZ, YZ projections	
f_16_, f_17_, f_18_: Length major axis of XY, XZ, YZ projections	f_37_, f_38_, f_39_: Hu moment 6 over XY, XZ, YZ projections	
f_19_, f_20_, f_21_: Length minor axis of XY, XZ, YZ projections	f_40_, f_41_, f_42_: Hu moment 7 over XY, XZ, YZ projections	

**Table 2:** kNN, NBC and SVM configuration parameters.

Configuration	kNN	NBC	SVM
Method	Euclidean, Mahalanobis	Gauss ^(2)^, KSF ^(3)^	Linear ^(4)^, quadratic ^(5)^
Data normalisation	Yes ^(1)^	No	Yes ^(1)^
Metrics	LOOCV, ROC	LOOCV, ROC	LOOCV, ROC
Classes	2	2	2

^(1)^ Normalised based on $(x_{i}-\mu )/\sigma$; ^(2)^ Kernel Smoothing Function: G(*x*) = ((2 * π)^−0.5^) * exp(−0.5 * *x*^2^); ^(3)^ Kernel Smoothing Function: $f_{h}\left(x\right)=\left(\frac{1}{n\;*\;h}\right)\sum_{i=1}^{n}K\left(\frac{x\;-\;x_{i}}{h}\right)$; ^(4)^ Kernel Smoothing Function: G(*x*_1_,*x*_2_) = *x*_1_’*x*_2_; ^(5)^ Kernel Smoothing Function: G(*x*_1_,*x*_2_) = (1 + *x*_1_’*x*_2_)^2^.

**Table 3:** LOOCV error and AUC for kNN, NBC, SVM.

MLA		kNN		NBC		SVM	
*Configuration*		*Euclidean*	*Mahalanobis*	*KSF*	*Gauss*	*Linear*	*Quadratic*
**LOOCV**	**Error**	0.0653	0.0673	0.1361	0.6769	**0.0528**	**0.0451**
**ROC curves**	**AUC**	**0.9935**	0.9916	0.9931	0.9317	0.9764	**1.0000**
	**Sensivity**	0.9764	0.9727	0.9304	0.2122	0.9758	1.0000
	**Specificity**	0.9205	0.9169	0.9891	0.9855	0.8194	1.0000
	**Precision**	0.9865	0.985907	0.9980	0.9887	0.9699	1.0000
	**Accuracy**	0.9684	0.964785	0.9388	0.3231	0.9533	1.0000
	**Fscore**	**0.9814**	0.9793	0.9630	0.3494	0.9728	**1.0000**

**Table 4:** TP, FP, TN and FN computed seven frames of scenario 3.

			kNN–Euclidean				SVM–Linear				SVM–Quadratic			
Frame	Samples	Pedestrians in the Sample	TP	FP	TN	FN	TP	FP	TN	FN	TP	FP	TN	FN
1	100	1	1	7	92	0	1	2	97	0	1	14	85	0
2	53	1	1	5	47	0	1	2	50	0	0	3	49	1
3	47	1	1	4	42	0	1	1	45	0	1	10	36	0
4	58	3	1	7	48	2	1	3	52	2	2	6	49	1
5	79	4	3	5	70	1	3	3	72	1	4	9	66	0
6	45	4	4	5	36	0	4	1	40	0	4	2	39	0
7	103	2	2	9	92	0	2	3	98	0	2	6	95	0
Sum	485	16	13	42	427	3	13	15	454	3	14	50	419	2

**Table 5:** Scenario metrics.

Metric	kNN–Euclidean	SVM–Linear	SVM–Quadratic
Sensivity	0.8125	0.8125	0.8750
Specificity	0.9104	0.9680	0.8934
Precision	0.2364	0.4643	0.2188
Accuracy	0.9072	0.9629	0.8928
Fscore	0.3662	0.5909	0.3500

**Table 6:** Comparison of proposed method with other authors.

Author/Year [Ref.]	MLA	Metric		Performance
		Graphical	Numeric	
Proposed 2016	Linear SVM	ROC curve	LOOCV, AUC, sensitivity, specificity, precision, accuracy, Fscore	0.0528, *0.9764*, 0.9758, 0.8194, 0.9699, 0.9533, *0.9728*
Premebida 2014 [18]	Deformable Part-based Model (DPM)	Precision-Recall curve of the areas of the pedestrian correctly identified.	Authors report a mean	0.3950
Spinello 2010 [19]	Multiple AdaBoost classifiers	Precision-Recall curve, Equal Error Rates (EER)	Authors report a mean	0.7760
Navarro-Serment 2010 [20]	Two SVMs in cascade	Precision-Recall and ROC curves	AUC estimate	0.8500
Ogawa 2011 [21]	Interacting Multiple Model filter	Recognition rate	Authors report a mean	0.8000
Kidono 2011 [22]	SVM	ROC curve	AUC estimate	0.9000

## Abbreviations

The following abbreviations are used in this manuscript:

ADAS	Advance Driver Assistance Systems
AUC	Area under curve
AV	Autonomous vehicles
CIC	Cloud Incubator Car
kNN	*k*-nearest neighbour
KSF	Kernel smoothing function
LOOCV	Leave-one-out cross validation method
MLA	Machine learning algorithms
NBC	Naïve Bayes classifier
SVM	Support vector machines

## References

[1] Thrun S. (2010). Toward robotic cars. Commun. ACM.

[2] IEEE Expert Members of IEEE Identify Driverless Cars as Most Viable Form of Intelligent Transportation, Dominating the Roadway by 2040 and Sparking Dramatic Changes in Vehicular Travel. http://www.ieee.org/about/news/2012/5september_2_2012.html.

[3] Fagnant D.J., Kockelman K. (2015). Preparing a nation for autonomous vehicles: Opportunities, barriers and policy recommendations. Transp. Res. Part A Policy Pract..

[4] Comission E. Statistics of Road Safety. http://ec.europa.eu/transport/road_safety/specialist/statistics/index_en.htm.

[5] 2015 Motor Vehicle Crashes: Overview. https://crashstats.nhtsa.dot.gov/Api/Public/ViewPublication/812318.

[6] Dollar P., Wojek C., Schiele B., Perona P. (2012). Pedestrian Detection: An Evaluation of the State of the Art. IEEE Trans. Pattern Anal. Mach. Intell..

[7] Benenson R., Omran M., Hosang J., Schiele B. (2015). Ten Years of Pedestrian Detection, What Have We Learned?. Computer Vision—ECCV 2014 Workshops.

[8] Zhao T., Nevatia R., Wu B. (2008). Segmentation and Tracking of Multiple Humans in Crowded Environments. IEEE Trans. Pattern Anal. Mach. Intell..

[9] Kristoffersen M., Dueholm J., Gade R., Moeslund T. (2016). Pedestrian Counting with Occlusion Handling Using Stereo Thermal Cameras. Sensors.

[10] Satake J., Chiba M., Miura J. (2013). Visual person identification using a distance-dependent appearance model for a person following robot. Int. J. Autom. Comput..

[11] Tsutsui H., Miura J., Shirai Y. Optical flow-based person tracking by multiple cameras. Proceedings of the International Conference on Multisensor Fusion and Integration for Intelligent Systems, MFI 2001.

[12] Dalal N., Triggs W. Histograms of Oriented Gradients for Human Detection. Proceedings of the IEEE Computer Society Conference on Computer Vision and Pattern Recognition (CVPR 2005).

[13] Szarvas M., Sakait U., Ogata J. Real-Time Pedestrian Detection Using LIDAR and Convolutional Neural Networks. Proceedings of the 2006 IEEE Intelligent Vehicles Symposium.

[14] Zhu Q., Chen L., Li Q., Li M., Nüchter A., Wang J. 3D LIDAR point cloud based intersection recognition for autonomous driving. Proceedings of the 2012 IEEE Intelligent Vehicles Symposium (IV).

[15] Arastounia M. (2016). Automated As-Built Model Generation of Subway Tunnels from Mobile LiDAR Data. Sensors.

[16] Hämmerle M., Höfle B. (2014). Effects of Reduced Terrestrial LiDAR Point Density on High-Resolution Grain Crop Surface Models in Precision Agriculture. Sensors.

[17] Yan L., Liu H., Tan J., Li Z., Xie H., Chen C. (2016). Scan Line Based Road Marking Extraction from Mobile LiDAR Point Clouds. Sensors.

[18] Premebida C., Batista J., Nunes U. Pedestrain Detection Combining RGB and Dense LiDAR Data. Proceedings of the 2014 IEEE/RSJ International Conference on Intelligent Robots and Systems (IROS 2014).

[19] Spinello L., Arras K., Triebel R., Siegwart R. A Layered Approach to People Detection in 3D Range Data. Proceedings of the AAAI Conference on Artificial Intelligence.

[20] Navarro-Serment L.E., Mertz C., Hebert M. (2010). Pedestrian Detection and Tracking Using Three-dimensional LADAR Data. Int. J. Robot. Res..

[21] Ogawa T., Sakai H., Suzuki Y., Takagi K., Morikawa K. Pedestrian detection and tracking using in-vehicle LiDAR for automotive application. Proceedings of the IEEE Intelligent Vehicles Symposium (IV).

[22] Kidono K., Miyasaka T., Watanabe A., Naito T., Miura J. Pedestrian recognition using high-definition LIDAR. Proceedings of the IEEE Intelligent Vehicles Symposium (IV).

[23] Toews M., Arbel T. Entropy-of-likelihood feature selection for image correspondence. Proceedings of the Ninth IEEE International Conference on Computer Vision.

[24] Chan C.-H., Pang G.K.H. (2000). Fabric defect detection by Fourier analysis. IEEE Trans. Ind. Appl..

[25] Chen L., Lu G., Zhang D.S. Effects of different gabor filter parameters on image retrieval by texture. Proceedings of the 10th International Multimedia Modeling Conference (MMM 2004).

[26] Fernández-Isla C., Navarro P.J., Alcover P.M. (2013). Automated Visual Inspection of Ship Hull Surfaces Using the Wavelet Transform. Math. Probl. Eng..

[27] Navarro P.J., Pérez F., Weiss J., Egea-Cortines M. (2016). Machine learning and computer vision system for phenotype data acquisition and analysis in plants. Sensors.

[28] Chen Y.Q., Nixon M.S., Thomas D.W. (1995). Statistical geometrical features for texture classification. Pattern Recognit..

[29] Haralick R.M., Shanmugam K., Dinstein I.H. (1973). Textural features for image classification. IEEE Trans. Syst. Man Cybern..

[30] Zucker S.W., Terzopoulos D. (1980). Finding structure in Co-occurrence matrices for texture analysis. Comput. Graph. Image Process..

[31] Aggarwal N., Agrawal R.K. (2012). First and Second Order Statistics Features for Classification of Magnetic Resonance Brain Images. J. Signal Inf. Process..

[32] Hu M.K. (1962). Visual pattern recognition by moment invariants. IRE Trans. Inf. Theory.

[33] Zhang Y., Wang S., Sun P. (2015). Pathological brain detection based on wavelet entropy and Hu moment invariants. Bio-Med. Mater. Eng..

[34] Alexa Internet. http://www.alexa.com/siteinfo/google.com.

[35] Murphy K.P. (2012). Machine Learning: A Probabilistic Perspective.

[36] Lantz B. (2013). Machine Learning with R.

[37] Navarro P.J., Alonso D., Stathis K. (2016). Automatic detection of microaneurysms in diabetic retinopathy fundus images using the L*a*b color space. J. Opt. Soc. Am. A. Opt. Image Sci. Vis..

[38] Bradley A.P. (1997). The use of the area under the ROC curve in the evaluation of machine learning algorithms. Pattern Recognit..

[39] Hand D. (2009). Measuring classifier performance: A coherent alternative to the area under the ROC curve. Mach. Learn..

